# Assessing the Predictive Power of PIRCHE-II Scores for the Development of *De Novo* Donor-Specific Antibodies After Simultaneous Pancreas-Kidney Transplantation

**DOI:** 10.3389/ti.2024.13720

**Published:** 2024-12-18

**Authors:** Francesca Raineri, Lukas Frischknecht, Jakob Nilsson, Fabian Rössler, Claudia Cavelti-Weder, Seraina von Moos, Thomas Schachtner

**Affiliations:** ^1^ Department of Nephrology, University Hospital Zurich, Zurich, Switzerland; ^2^ Department of Immunology, University Hospital Zurich, Zurich, Switzerland; ^3^ Department of Surgery and Transplantation, University Hospital Zurich, Zurich, Switzerland; ^4^ Department of Endocrinology, Diabetology and Clinical Nutrition, University Hospital Zurich, Zurich, Switzerland; ^5^ Department of Nephrology, Canton Hospital Luzern, Lucerne, Switzerland

**Keywords:** simultaneous pancreas and kidney transplant, preformed DSA, *de novo* DSA, epitope matching, PIRCHE-II score

## Abstract

The molecular HLA epitope mismatch is an advanced measure for developing *de novo* donor-specific antibodies (dnDSA) after kidney transplantation. Its relevance in simultaneous pancreas/kidney transplant recipients (SPKTRs) remains unclear. We investigated dnDSA development in 72 SPKTRs and 383 kidney transplant recipients (KTRs) and used the Predicted Indirectly Recognizable HLA-Epitopes (PIRCHE-II) algorithm to calculate the mismatch load of HLA-derived epitopes in total, per HLA-class, and per HLA-locus. At 1 year post-transplant, SPKTRs exhibited an increased dnDSA incidence (11.2% vs. 3.1%, *p* = 0.011); but not at 10 years post-transplant. In SPKTRs, preformed DSA (HR 2.872, *p* = 0.039) and younger donor age (HR 0.943, *p* = 0.017) were independent risk factors for developing dnDSA. PIRCHE-II scores for HLA-DQ correlated with dnDSA development upon univariate analysis (*p* = 0.044). Among 455 KTRs/SPKTRs, multivariate analysis identified PIRCHE-II scores for HLA-DQ (HR 1.023, *p* = 0.025) and ciclosporine use (HR 2.440, *p* = 0.001) as independent predictors of dnDSA development. Simultaneous pancreas/kidney transplantation (SPK) was an independent risk factor in case of preformed DSA only (HR 2.782, *p* = 0.037). High PIRCHE-II scores for HLA-DQ are crucial for dnDSA development in both SPKTRs and KTRs. The lack of an independent association of total PIRCHE-II scores urges caution in implementing it in post-transplantation risk assessment.

## Graphical Abstract

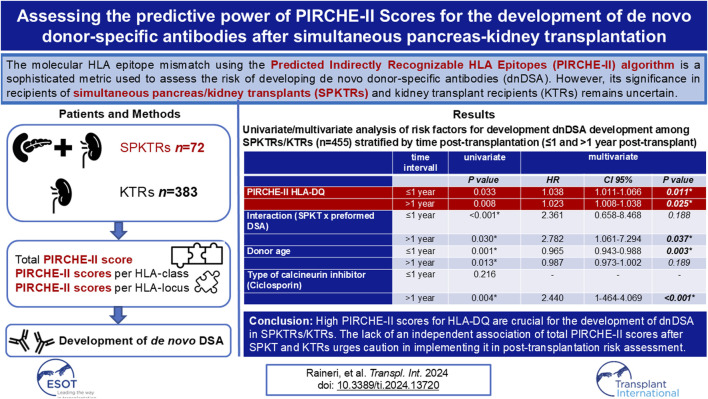

## Introduction

In June 2021, the First World Consensus Conference on Pancreas Transplantation provided evidence-based guidelines, offering directions for clinical practice after pancreas transplantation (PT) [1, 2]. The primary message emphasized that both simultaneous pancreas/kidney transplantation (SPK) and pancreas transplantation alone (PTA) lead to improved quality of life [3, 4] and long-term patient survival [5, 6] compared to other medical interventions. Experts conclude that according to empirical evidence, preformed donor-specific anti-HLA antibodies (DSA) with MFI level <5000 in recipients with negative T and B cell flow cytometric crossmatches should not be a prohibitive factor for pancreas transplantation, based on the fact that evidence regarding negative impact of pretransplant DSA on transplant outcomes is lacking [2]. Contrarily, detection of dnDSA has been associated with worse outcomes, including graft rejection and failure [7, 8], Hence, rigorous post-transplant surveillance is recommended. In the setting of SPK, the relevance of HLA mismatching is a matter of debate as it did not translate into improved overall graft outcome, even though associated with reduced development of dnDSA and reduced graft rejection [9, 10]. Concerning immunosuppression, tacrolimus and mycophenolate, compared to ciclosporin and azathioprine, showed superior immunological results, i.e., reduced risk of developing dnDSA [11, 12]. Early tapering of corticosteroids was found suitable for a specific subset of pancreas transplant recipients, demonstrating viability without concomitant compromise in outcomes but improved metabolic parameters in the long term [13, 14].

Our study aims to assess the impact of the Predicted Indirectly ReCognizable HLA Epitopes (PIRCHE-II) scores for the first time in predicting the development of dnDSA and graft outcomes in simultaneous pancreas/kidney transplantation recipients. The PIRCHE-II score is an established algorithm to calculate HLA epitope mismatches for certain HLA antigen mismatches. It estimates the number of indirectly recognizable, donor HLA-derived T cell epitopes and predicts T cell-related immune responses against the donor HLA-derived peptides. Moreover, the PIRCHE-II score has demonstrated the ability to predict the incidence of dnDSA in kidney transplantation (KT) independently and was associated with kidney allograft survival in a cohort of kidney transplantation [15, 16].

In our study, we attempted to address the following questions: 1) What is the incidence of dnDSA among SPKTRs vs. KTRs? 2) What risk factors are associated with the development of dnDSA among SPKTRs at 1-year post-transplantation and in the long-term? 3) What risk factors are associated with the development of dnDSA among the whole cohort of SPKTRs/KTRs at 1-year post-transplantation and in the long term?

## Materials and Methods

### Patients

Our study was approved by the Cantonal Ethic Commission Review Board of Zurich, Switzerland (KEK-ZH Number 2020-02817) and has complied with the Declaration of Helsinki.

We conducted a retrospective study of 72 SPKTRs who underwent a first deceased donor SPK and 383 KTRs who underwent a first deceased-donor single kidney transplantation at the University Hospital of Zurich between May 2009 and December 2019. Allograft outcome was evaluated in terms of 1) kidney allograft function, survival, and graft rejection, 2) pancreas allograft function, survival, and graft rejection, and 3) the development of dnDSA.

Post-transplant care was carried out according to a standardized scheme with appointments in our outpatient clinic twice a week at weeks 2 and 3, once a week at weeks 4, 5, 6, 8, 10, 12, once a month at months 4, 5, 6, 8, 10 and 12, with at least 16 visits within the first year after transplant. Subsequently, quarterly check-ups were performed in cooperation with the nephrologists close to the patient’s home, with at least annual follow-up visits in our outpatient clinic. At any appointment, kidney function was evaluated by measuring serum creatinine, serum urea, and proteinuria. Pancreas graft function was defined as insulin-free survival and was assessed by the measurement of serum lipase and fasting plasma glucose levels. In addition, HbA1c values were routinely checked at the first visit, at week 12, at months 6, 9, and 12, annually, and at any time pancreas dysfunction was suspected.

### Induction and Maintenance Immunosuppression

Among both SPKTRs and KTRs, a peak MFI cut-off of 1,000 of any historic preformed DSA was applied for acceptance of an organ offer. All 72 SPKTRs received lymphocyte-depleting induction immunosuppression. The maintenance immunosuppression consisted of a dual-drug combination of a calcineurin inhibitor (CNI, tacrolimus) and antimetabolite (MMF, mycophenolate mofetil) or (MPA, mycophenolic acid) or azathioprine. Early steroid withdrawal within the first post-transplant week was performed in all SPKTRs.

Regarding the individually defined immunologic risk, 383 KTRs received lymphocyte-depleting induction or induction with interleukin-2 receptor blockade. The primary immunosuppression consisted of a dual-drug combination of a calcineurin inhibitor (CNI, tacrolimus, or ciclosporin) and antimetabolite (MMF, mycophenolate mofetil) or (MPA, mycophenolic acid) or azathioprine, and steroids. Steroids were reduced over 12 weeks to 5 mg prednisone/day. KTRs underwent steroid withdrawal at +6 months post-transplantation unless 1) preformed DSA persisted with an MFI >500, 2) dnDSA developed with an MFI >500, or 3) KTRs had glomerulonephritis as the underlying disease.

### Assessment of Kidney and Pancreas Allografts Function and Survival

Kidney allograft function, survival, and rejection were evaluated based on the best serum creatinine (µmol/L), best proteinuria (mg/day), and eGFR (mL/min) at 1 year post-transplant. The best serum creatinine and best proteinuria were calculated as the median of the 3 lowest serum creatinine and proteinuria values in the first post-transplant year. Additionally, kidney graft outcomes were evaluated based on the need for re-transplant, dialysis treatment, or patient death.

Pancreas allograft function, survival, and rejection were evaluated based on the need for insulin therapy, best HbA1c value in the first 2 years post-transplant, and the need for pancreas re-transplant or patient death. The best HbA1c value was calculated as the median of the 3 lowest HbA1c values in the 1- and 2-years post-transplant.

### HLA Typing, Anti-HLA Antibody Analysis and Calculation of Predicted Indirectly ReCognizable HLA Epitopes (PIRCHE-II)

The HLA-derived mismatched peptide epitopes presented by SPKTRs HLA-molecules were calculated using the PIRCHE-II algorithm. In addition to the standard donor HLA typing, further typing was performed to assess additional loci if the recipient developed anti-HLA antibodies after transplantation against an HLA locus that had not been previously typed. For each HLA locus, the presentation of both HLA class I (HLA-A, B, C) and HLA class II-derived peptides (HLA-DR, DQ) were calculated and designated PIRCHE-II-A, B, C, DR, and DQ, respectively. HLA typing of donors and recipients was determined using either sequence-specific oligonucleotide (SSO), sequence-specific primer (SSP), or Next-generation sequencing (NGS) technologies depending on when they were transplanted. For all PIRCHE-II calculations only low-resolution HLA typing was entered and the high-resolution typing was imputed according to the standard PRICHE-II algorithm. The PIRCHE-II algorithm is available online.^1^ For class I scores, the PIRCHE-II score is the sum of HLA-A, HLA-B, and HLA-C scores, while for class II scores, it is the sum of HLA-DRB1 and HLA-DQB1 scores. The total PIRCHE-II score is the sum of all loci scores for each donor-recipient pair.

The anti-HLA antibodies testing was routinely performed with the use of a Luminex-based single bead assay (One Lambda, Canoga Park, CA, United States) on the day of the transplant, at months 3, 6, 12, annually after that, and at any other time in case of unexplained deterioration of allografts function. Positivity of dnDSAs were defined by the presence of dnDSA targeting the HLA loci A, B, C, DRB (including DRB345), DQB and DPB with a normalized mean fluorescence intensity (MFI) exceeding 500. The dnDSA detected post-transplant were analyzed individually by a specialist in transplantation immunology in a blinded fashion. Here, it was determined if the antibody showed true donor specificity by analyzing the pattern of single-bead reactivity and comparing it to the HLA typing of the donor.

SPKTRs/KTRs with 0 HLA-antigen mismatches, 0 HLA-antigen mismatches for HLA-class I, and 0 HLA-antigen mismatches for HLA-classes II were excluded for the distinct analyses.

### Statistical Methods

Clinical characteristics are expressed as numbers (%) and were compared across groups using Fisher’s exact test for categorical variables. Continuous variables are expressed as median (range: minimum-maximum) and were compared using Mann Whitney-U Test. Statistical analysis was performed using IBM SPSS Version 28.0.1.1. Survival was analyzed using the Kaplan-Meier method and compared with the LogRank test. Univariable and multivariable Cox proportional hazards models with the enter method were used to investigate factors associated with survival. Bonferroni adjustment was applied to account for multiple comparisons, restricting the correction to the analyses involving the different PIRCHE-II scores. Variables with a *p*-value ≤0.05 in the univariable analysis were included in the multivariable model. Statistical significance was assumed for a two-tailed *p*-value <0.05 for all tests.

## Results

### Overall Patient Characteristics

Tables 1, 2 shows the clinical characteristics and outcomes of SPKTRs and KTRs. In the SPKTR cohort, all patients underwent thymoglobulin induction immunosuppressive therapy and received tacrolimus as maintenance calcineurin inhibitor therapy. Moreover, none of the SPKTR patients received prednisone for maintenance therapy. Additionally, the donors’ age in the SPKTR cohort was significantly younger than in the KTRS cohort.

**TABLE 1:** Clinical characteristics of SPKTRs and KTRs at transplantation.

	Total (n = 455)	SPKTRs (n = 72)	KTRs (n = 383)	*P*-value
Recipient characteristics				
Time post-transplant, months*	70 (6–158)	74 (6–158)	68 (11–157)	0.349
Recipient age, years*	53 (17–75)	43 (23–58)	55 (17–75)	<0.001*
Recipient, male sex, n (%)	278 (62)	36 (50)	242 (63)	0.048*
Underlying kidney disease, n (%) Type 1 diabetes Type 2 diabetes Other	70 (15) 29 (6) 356 (78)	68 (94) 3 (4) 1 (1)	2 (1) 26 (7) 355 (93)	<0.001* 0.598 <0.001*
BMI pre-transplant, kg/m^2^	25 (16.44–41.21)	24 (16.95–34.14)	25 (16.44–41.21)	<0.001*
Deceased donation, n (%)	455 (100)	72 (100)	383 (100)	1
Cold ischemia time h:min*	9 h 27 min (567 min)	9 h 54 min (594 min)	9 h 12 min (554 min)	0.239
Induction IS, n (%) Lymphocyte depletion IL-2 receptor blockade	189 (42) 266 (58)	72 (100) 0 (0)	117 (31) 266 (69)	<0.001* <0.001*
Maintenance IS, n (%) Tacrolimus Everolimus Ciclosporin MMF EC-MPA Azathioprine	396 (87) 1 (0) 58 (13) 372 (82) 81 (18) 2 (0)	72 (100) 0 (0) 0 (0) 22 (31) 49 (67) 1 (1)	324 (85) 1 (0) 58 (15) 350 (91) 32 (8) 1 (0)	<0.001* 1 <0.001* <0.001* <0.001* 0.291
Donor Characteristics				
Donor age, years*	52 (10–88)	34 (11–57)	55 (10–88)	<0.001*
Donor male sex, n (%)	268 (59)	53 (74)	215 (53)	0.006*
Immunocompatibility				
Total HLA Mismatches *	6 (0–10)	6 (2–10)	5 (0–10)	<0.001*
Total PIRCHE-II Score* PIRCHE-II A* PIRCHE-II B* PIRCHE-II C* PIRCHE-II HLA I PIRCHE-II DR* PIRCHE-II DQ* PIRCHE-II HLA II	71.32 (0–233.55) 14.95 (0–62.52) 14.72 (0–54.19) 12.63 (0–75.06) 13.85 (0–75.06) 12.00 (0–56.13) 19.00 (0–80.60) 14.16 (0–80.60)	60.495 (20.63–165.83) 10.56 (0–48.59) 11.16 (0.05–35.93) 11.65 (0–50.00) 11.32 (0–50.00) 11.26 (0–46.51) 17.05 (0–52.84) 14.07 (0–52.84)	73.47 (0–233.55) 15.63 (0–62.52) 15.09 (0–54.19) 13.00 (0–75.06) 14.62 (0–75.06) 12.00 (0–56.13) 19.30 (0–80.60) 14.20 (0–80.60)	0.009* 0.008* 0.088 0.059 0.059 0.999 0.268 0.237
Preformed DSA, n (%)^a^	139 (31)	16 (22)	123 (32)	0.124
HLA-A HLA-B HLA-Cw HLA-DRB HLA-DR51-53 HLA-DQB HLA-DP	42 (9) 31 (7) 25 (5) 39 (9) 30 (7) 56 (12) 9 (2)	3 (4) 3 (4) 1 (1) 2 (3) 5 (7) 9 (13) 0 (0)	39 (10) 28 (7) 24 (6) 37 (10) 25 (7) 47 (12) 9 (2)	0.122 0.448 0.153 0.064 0.800 1 0.366
Persistence of preformed DSA after transplantation	74 (53)	13 (81)	61 (50)	0.606
HLA-A HLA-B HLA-Cw HLA-DRB HLA-DR51-53 HLA-DQB HLA-DP	6 (1) 2 (0) 11 (2) 9 (2) 14 (3) 30 (7) 2 (0)	1 (1) 0 (0) 1 (1) 1 (1) 3 (4) 7 (10) 0 (0)	5 (1) 2 (1) 10 (3) 8 (2.) 11 (3) 23 (6) 2 (1)	1 1 1 0.566 0.472 0.296 1
Maximum peak of preformed DSA after transplantation*	—	1,610 (865–25,767)	1,353 (551–8,173)	—
HLA-A* HLA-B* HLA-Cw* HLA-DRB* HLA-DR51-53* HLA-DQB* HLA-DP*	— — — — — — —	2,648 (600–2,855) 1,092 (640–1,545) 5007 (5,007–5,007) 1,352 (1,352–1,352) 3,070 (1,448–4,089) 1,610 (865–25,767) 0	1,353 (509–10,299) 1904 (551–7,386) 1,060 (518–5,348) 1,009 (563–4,179) 1,154 (682–16,815) 1820 (516–21,358) 2,285 (852–8,173)	— — — — — — —

*median (range).

^a^ Preformed DSA: DSA against the current kidney/pancreas allograft with MFI>500 at any time before transplantation. Each percentage refers to the total number of patients in the SPKTRs/KTRs cohort. No cases of either preformed DSA, directed against HLA-DQA or HLA-DPA were identified among SPKTRs and KTRs.

**TABLE 2:** Outcomes of SPKTRs and KTRs.

	Total (455)	SPKTRs (n = 72)	KTRs (383)	*P*-value
Pancreas allograft function/survival				
Pancreas allograft loss/use of insulin, n (%)	12 (3)	12 (17)	—	—
Time to pancreas loss, months*		6		
Cause of pancreas allograft loss Thrombosis, n (%) Leakage, n (%) Steroid-induced diabetes mellitus, n (%) Others/unknown, n (%)	6 (1) 11 1 (0) 1 (0) 4 (1)	6 (8) 1 (1) 1 (1) 4 (6)	— — —	— — — —
Pancreas retransplantation, n (%)	4 (1)	4 (6)	—	—
History of HbA1c, %				
1 year post-transplant *	—	5.3	—	—
2 years post-transplant *	—	5.3	—	—
Best value since transplantation *	—	4.9	—	—
BMI post-transplant, kg/m^2^	25 (14.97–43.31)	23 (14.97–34.66)	—	—
Kidney allograft function/survival				
Kidney allograft loss, n (%) dialysis treatment patient’s death Kidney retransplantation graft survival after the first transplantation	22 (5) 68 (15) 73 (16) 380 (84)	1 (1) 4 (6) 2 (3) 70 (97)	21 (5) 64 (17) 71 (19) 310 (81)	0.226 0.011* <0.001* <0.001*
Baseline creatinine 1-year post-transplant, µmol/L *	117 (48–626)	98.5 (57–380)	120 (48–626)	<0.001*
Baseline proteinuria 1-year post-transplant, mg/day *	83 (0–1,693)	83 (0–780)	83 (0–1,693)	0.394
eGFR (CKD-Epi) at 1-year post-transplant, mL/min *	55 (6–120)	67 (15–116)	52 (6–120)	<0.001*
Rejection in KTRs biopsy, n (%)	91 (20)	11 (15)	80 (21)	0.336
TCMR, n (%) ABMR, n (%)	57 (13) 34 (7)	7 (10) 4 (6)	50 (13) 30 (8)	0.561 0.630
Steroid free at 1 year, n (%)	242 (53)	51 (78)	191 (50)	0.0012*
*De novo* DSA, n (%)^a^	75 (16)	16 (22)	59 (15)	
HLA-A HLA-B HLA-Cw HLA-DRB HLA-DR51-53 HLA-DQB HLA-DP	14 (3) 17 (3) 3 (1) 27 (4) 15 (3) 48 (11) 6 (1)	2 (3) 5 (7) 0 (0) 6 (8) 4 (6) 12 (17) 2 (3)	12 (3) 12 (3) 3 (1) 21 (5) 11 (3) 36 (9) 4 (1)	— — — — — — —
Peak of *de novo* DSA after transplantation*	1,638 (514–17,553)	1,550 (548–14,458)	2,188 (514–17,553)	—
HLA-A* HLA-B* HLA-Cw* HLA-DRB* HLA-DR51-53* HLA-DQB* HLA-DP*	— — — — — — —	573 (511–788) 1,443 (505–3,345) — 796 (527–1,708) 682 (549–1,408) 1,383 (510–14,458) 1,076 (528–1,551)	1,030 (551–9,549) 809 (507–8,760) 2,359 (724–4,527) 762 (501–2,520) 746 (515–16,082) 1,867 (549–17,553) 955 (501–3,902)	— — — — — — —

*median (range).

^a^ *De novo* DSA: DSA against the current kidney/pancreas allograft with MFI>500 at any time before transplantation. Each percentage refers to the total number of patients in the SPKTRs/KTRs cohort. *De novo* DSA against HLA-DQA were detected in 1 and 3 SPKTRs and KTRs, respectively, while *de novo* DSA against HLA-DPA were found in 0 and 1 SPKTRs and KTRs, respectively.

**FIGURE 1:**
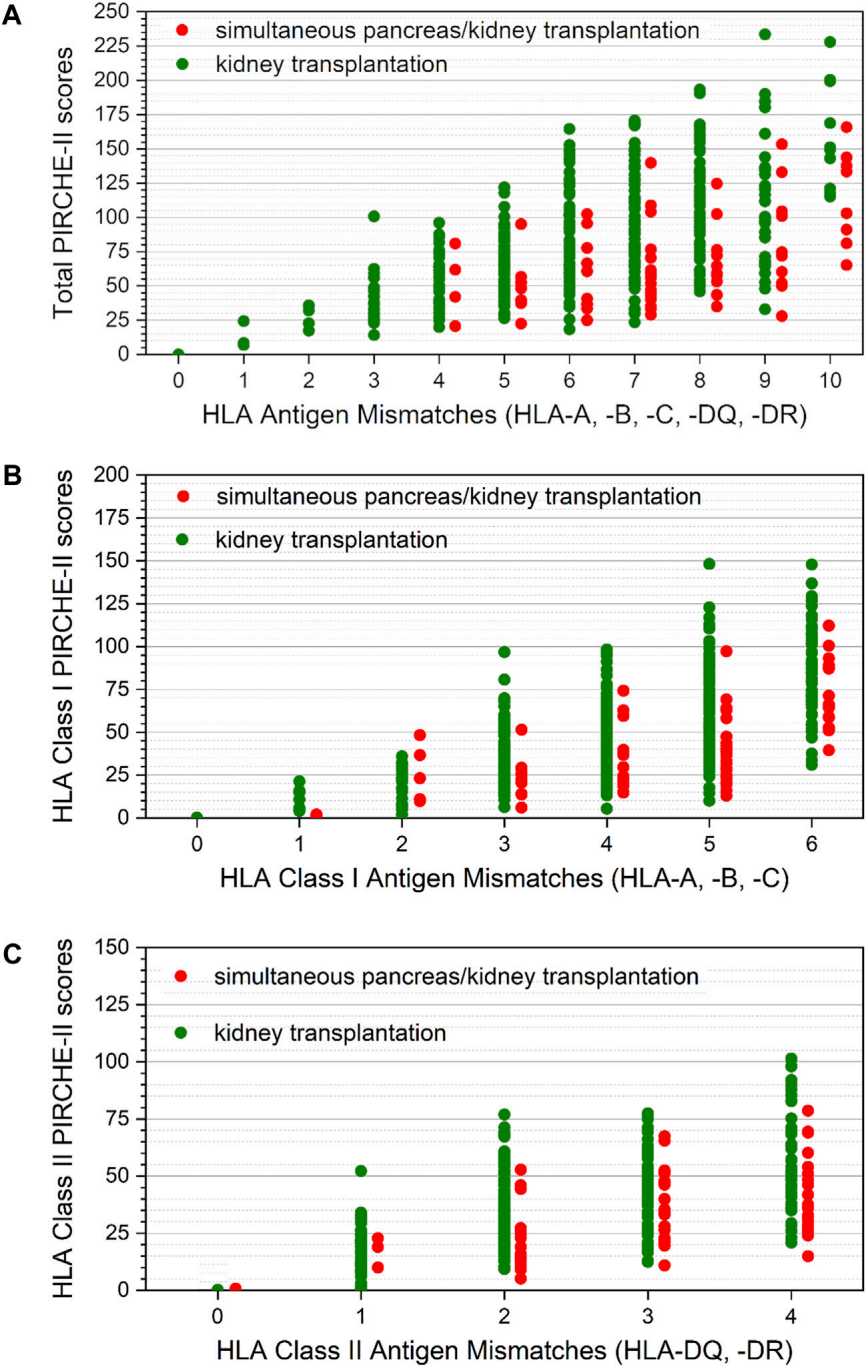
**(A)** Distribution of total PIRCHE-II scores compared to total HLA-mismatches. Total PIRCHE-II scores and the number of HLA mismatches were calculated from HLA class I (HLA-A, B, C) and HLA class II (HLA-DQ, DR) mismatches. Median PIRCHE-II scores for SPKTRs (red) and KTRs (green) were 60.25 (IQR 43.29–92.08) and 73.19 (IQR 53.47–107.25), respectively. **(B)** Distribution of total PIRCHE-II scores for HLA-class I antigens compared to HLA-class I mismatches. PIRCHE-II scores for HLA-class I and the number of HLA-class I mismatches were calculated from HLA-class I (HLA-A, B, C) mismatches. Median PIRCHE-II scores for HLA-class I for SPKTRs (red) and KTRs (green) were 36.59 (IQR 21.64–58.75) and 43.76 (IQR 28.73–68.65), respectively. **(C)** Distribution of total PIRCHE-II scores for HLA-class II antigens compared to HLA-class II mismatches. PIRCHE-II scores for HLA-class II and the number of HLA-class II mismatches were calculated from HLA-class II (HLA-DQ, DR) mismatches. Median PIRCHE-II scores for HLA-class I for SPKTRs (red) and KTRs (green) were 26.92 (IQR 18.22–40.45) and 31.26 (IQR 18.16–47.38), respectively.

The median total PIRCHE-II score of SPKTRs was 60.495 (range: 20.63–165.83), with PIRCHE-II for HLA-class I antigens of 36.56 (1.02–112.17) and PIRCHE-II for HLA-class II antigens of 26.92 (0–78.56; Figures 1A–C). Preformed DSA were detected in 16/72 (22%) SPKTRs, of which 9/72 (12.5%) SPKTRs showed preformed DSA against HLA-DQ.

The median total PIRCHE-II scores of KTRs was 73.47 (range: 0–233.55), with PIRCHE-II for HLA-class I antigens of 44.00 (0–148.23) and PIRCHE-II for HLA-class II antigens of 31.53 (0–101.82; Figures 1A–C). Preformed DSA were defined as DSA against graft(s) with MFI >500 at any time before transplantation. Preformed DSA were detected in 123 of 383 (32%) KTRs, of which 47 of 383 (12%) KTRs showed preformed DSA against HLA-DQ.

The median total PIRCHE-II score and the median PIRCHE-II score for HLA-class I antigens significantly differed between SPKTRs and KTRs (respectively, *p* = 0.009 and *p* < 0.001), while no significant difference was detected for PIRCHE-II Score for HLA class II antigens (*p* = 0.526).

### Graft Outcome

During the observation period of 10 years, 4 of 72 (6%) SPKTRs died, 12 of 72 (17%) SPKTRs lost their pancreas allograft function, and 1 of 72 (1%) SPKTRs returned to dialysis. During the observation period of 10 years, 64 of 383 (17%) KTRs died, and 21 of 383 (5%) KTRs returned to dialysis, being not significantly different as compared to SPKTRs (*p* = 0.226). Data about the development of TCMR and ABMR between SPKTRs and KTRs are shown in Supplementary Figures 1A, B.

### Development of dnDSA in SPKTRs and KTRs

**FIGURE 2:**
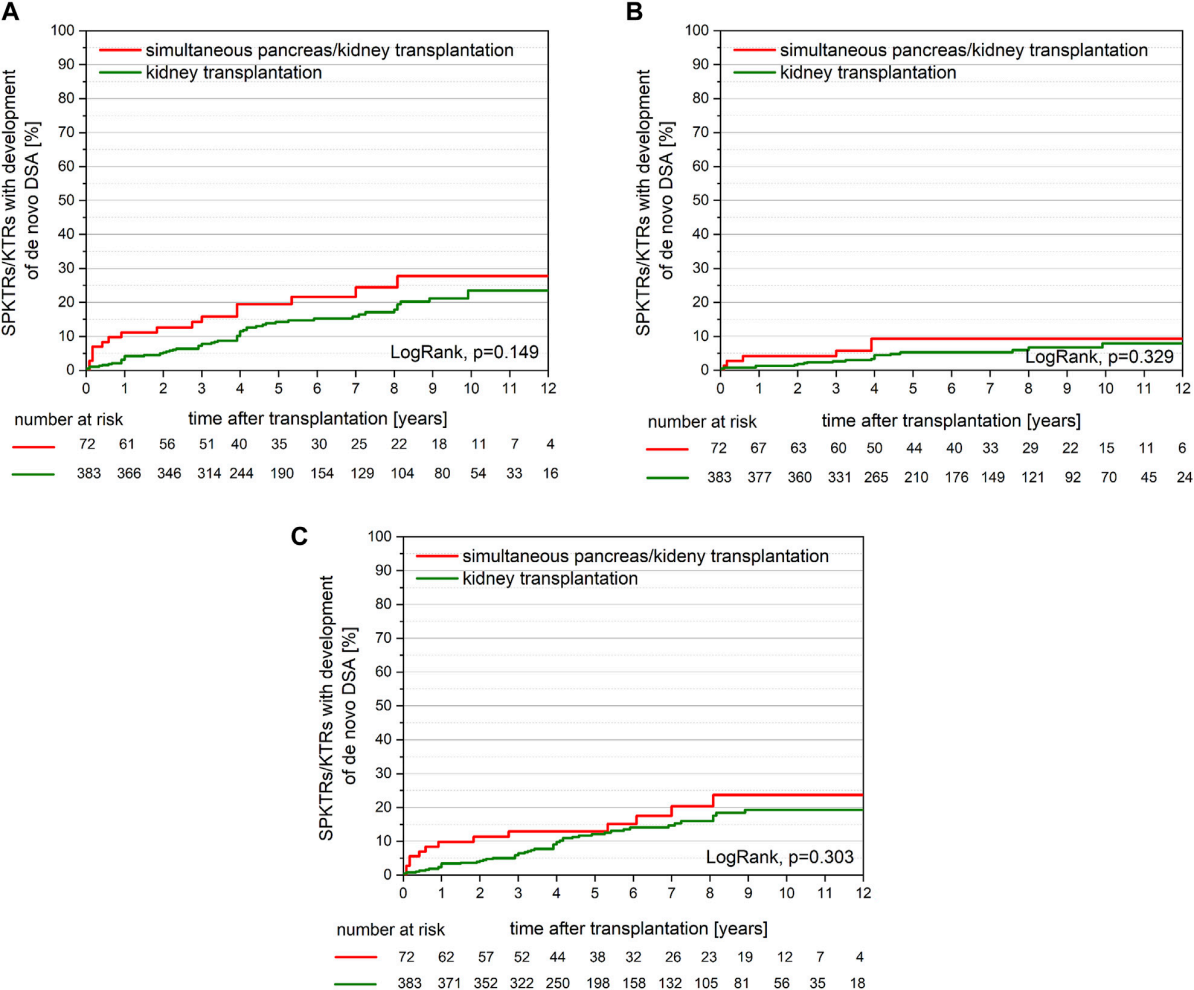
**(A)** Development of *de novo* DSA was comparable between SPKTRs (red) and KTRs (green; *p* = 0.149) with 27.7% vs. 23.5% at 10 years post-transplant, respectively. Interestingly, *de novo* DSA at 1-year post-transplantation were detectable in 11.2% of SPKTRs vs. 3.1% of KTRs (*p* = 0.011). **(B)** Development of *de novo* DSA against HLA-class I was comparable between SPKTRs (red) and KTRs (green; *p* = 0.329) with 9.3% vs. 8.0% at 10 years post-transplant, respectively. *De novo* DSA at 1 year post-transplantation were detectable in 4.2% of SPKTRs vs. 1.3% of KTRs (*p* = 0.086). **(C)** Development of *de novo* DSA against HLA-class II was comparable between SPKTRs (red) and KTRs (green; *p* = 0.303) with 23.7% vs. 19.3% at 10 years post-transplant, respectively. Interestingly, *de novo* DSA at 1-year post-transplantation were detectable in 9.8% of SPKTRs vs. 3.4% of KTRs (*p* = 0.012).

Overall, SPKTRs showed a trend towards a higher incidence of dnDSA compared to KTRs over the whole observation period. 16/72 (22%) SPKTRs developed dnDSA (Table 2) as compared to 59 of 383 (15%) KTRs. Yet, within the first year post-transplantation 8/72 (11%) SPKTRs developed dnDSA as compared to 16/383 (4%) SPKTRs/KTRs (*p* = 0.011; Figure 2A). Both dnDSA directed against HLA-class I (4% vs. 1%, *p* = 0.086) and HLA-class II dnDSA (10% vs. 3%, *p* = 0.012) were more frequently observed in SPKTRs as compared to KTRs in the first post-transplant year (Figures 2B, C). However, this difference did only reach statistical significance for dnDSA directed against HLA-class II.

### Risk Factors for the Development of dnDSA in SPKTRs

In our univariate analysis, PIRCHE-II scores per HLA locus, per HLA class, and total PIRCHE-II scores did not show an association with the development of dnDSA at 1 year post-transplantation or throughout the entire study period (Tables 3, 4). Conversely, the presence of preformed DSA significantly increased the risk of developing dnDSA both at 1 year post-transplantation (HR 4.432, CI 0.975–20.137, *p* = 0.054) and over the entire study period (HR 2.872, CI 1.053–7.831, *p* = 0.039). Additionally, a younger donor age was associated with a higher incidence of dnDSA over the study period (HR 0.943, CI 0.899–0.990, *p* = 0.017).

**TABLE 3:** Univariate and multivariate analysis of risk factors for the development of dnDSA at 1-year post-transplantation among SPKTRs (n = 72).

	Univariate	Multivariate		
	*P*-value	HR	CI 95%	*P*-value
PIRCHE-II HLA-A^§^	0.712	—	—	—
PIRCHE-II HLA-B^§^	0.158	—	—	—
PIRCHE-II HLA-C^§^	0.913	—	—	—
PIRCHE-II HLA-class I^§^	0.506	—	—	—
PIRCHE-II HLA-DR^§^	0.572	—	—	—
PIRCHE-II HLA-DQ^§^	0.655	—	—	—
PIRCHE-II HLA-class II^§^	0.559	—	—	—
Total PIRCHE-II^§^	0.403	—	—	—
Preformed DSA	0.017*	4.432	0.975–20.137	0.054
Recipient age	0.439	—	—	—
Donor age	0.100	0.969	0.907–1.034	0.339

^§^ *P*-values were adjusted for multiple comparisons using the Bonferroni correction, with a corrected significance level of 0.0056 (0.05/8) applied to the analyses involving the different PIRCHE-II scores. *P*-values ≤ 0.00625 are considered statistically significant.

**TABLE 4:** Univariate and multivariate analysis of risk factors for the development of dnDSA among SPKTRs overall (n = 72).

	Univariate	Multivariate		
	*P*-value	HR	CI 95%	*P*-value
PIRCHE-II HLA-A	0.241	—	—	—
PIRCHE-II HLA-B	0.249	—	—	—
PIRCHE-II HLA-C	0.399	—	—	—
PIRCHE-II HLA-class I	0.236	—	—	—
PIRCHE-II HLA-DR	0.246	—	—	—
PIRCHE-II HLA-DQ	0.284	—	—	—
PIRCHE-II HLA-class II	0.211	—	—	—
Total PIRCHE-II	0.179	—	—	—
Preformed DSA	0.001*	**2.872**	**1.053–7.831**	0.039*
Recipient age	0.208	—	—	—
Donor age	0.004*	**0.943**	**0.899–0.990**	0.017*

^§^ *P*-values were adjusted for multiple comparisons using the Bonferroni correction, with a corrected significance level of 0.0056 (0.05/8) applied to the analyses involving the different PIRCHE-II scores. *P*-values ≤ 0.00625 are considered statistically significant.

* statistically significant.

Similarly, PIRCHE-II scores per HLA locus, per HLA class, and total PIRCHE-II scores were not linked to the development of dnDSA against HLA class I in the univariate analysis (Table 5). However, in the univariate analysis, PIRCHE-II scores per HLA locus DQ were associated with an increased risk of developing dnDSA against HLA class II (*p* = 0.044). Multivariate analysis revealed that only preformed DSA remained independently associated with an increased risk of developing dnDSA against HLA class II (HR 4.700, CI 1.397–15.811, *p* = 0.012, Table 6).

**TABLE 5:** Univariate and multivariate analysis of risk factors for the development of dnDSA against HLA-class I among SPKTRs (n = 72).

	Univariate	Multivariate		
	*P*-value	HR	CI 95%	*P*-value
PIRCHE-II HLA-A	0.318	—	—	—
PIRCHE-II HLA-B	0.276	—	—	—
PIRCHE-II HLA-C	0.900	—	—	—
PIRCHE-II HLA-class I	0.241	—	—	—
PIRCHE-II HLA-DR	0.609	—	—	—
PIRCHE-II HLA-DQ	0.830	—	—	—
PIRCHE-II HLA-class II	0.448	—	—	—
Total PIRCHE-II	0.699	—	—	—
Preformed DSA	0.688	—	—	—
Recipient age	0.577	—	—	—
Donor age	0.655	—	—	—

^§^ *P*-values were adjusted for multiple comparisons using the Bonferroni correction, with a corrected significance level of 0.0056 (0.05/8) applied to the analyses involving the different PIRCHE-II scores. *P*-values ≤ 0.00625 are considered statistically significant.

**TABLE 6:** Univariate and multivariate analysis of risk factors for the development of dnDSA against HLA-class II among SPKTRs (n = 72).

	Univariate	Multivariate		
	*P*-value	HR	CI 95%	*P*-value
PIRCHE-II HLA-A	0.825	—	—	—
PIRCHE-II HLA-B	0.603	—	—	—
PIRCHE-II HLA-C	0.244	—	—	—
PIRCHE-II HLA-class I	0.907	—	—	—
PIRCHE-II HLA-DR	0.096	0.998	0.936–1.064	0.947
PIRCHE-II HLA-DQ	0.044*	1.040	0.989–1.094	0.124
PIRCHE-II HLA-class II	0.034*	—	—	—
Total PIRCHE-II	0.301	—	—	—
Preformed DSA	0.001*	**4.700**	**1.397–15.811**	0.012*
Recipient age	0.252	—	—	—
Donor age	0.015*	0.963	0.914–1.014	0.152

^§^ *P*-values were adjusted for multiple comparisons using the Bonferroni correction, with a corrected significance level of 0.0056 (0.05/8) applied to the analyses involving the different PIRCHE-II scores. *P*-values ≤ 0.00625 are considered statistically significant.

* statistically significant.

### Risk Factors for the Development of dnDSA in SPKTRs/KTRs

Among the whole cohort SPKTRs/KTRs multivariate analysis revealed that PIRCHE-II scores for HLA locus DQ and younger donor age were significantly associated with the development of dnDSA at 1 year post-transplantation (HR 1.038, CI 1.0011–1.066, *p* = 0.011; HR 0.965, CI 0.943–0.988, *p* = 0.003) and HLA locus DQ was significantly associated with the development of dnDSA after 1 year post-transplantation (HR 1.023, CI 1.008–1.038, *p* = 0.025) (Table 7). Additionally, using ciclosporin for maintenance immunosuppression was associated with an increased risk of developing dnDSA after 1 year post-transplantation (HR 2.440, CI 1.464–4.069, *p* < 0.001). Simultaneous pancreas-kidney transplantation (SPK) was not associated with dnDSA development in the multivariate analysis. However, SPK and the presence of preformed DSA independently increased the risk for the development of dnDSA after 1-year post-transplantation (HR 2.782, CI 1.061–7.294, 0.037).

**TABLE 7:** Univariate and multivariate analysis of risk factors for the development of dnDSA among SPKTRs/KTRs (n = 455) stratified by time post-transplantation (≤1 year post-transplant and >1 year post-transplant).

	Time interval	Univariate	Multivariate		
		*P*-value	HR	CI 95%	*P*-value
PIRCHE-II HLA-A^§^	≤1 year	0.350	—	—	—
	>1 year	0.125	—	—	—
PIRCHE-II HLA-B^§^	≤1 year	0.169	—	—	—
	>1 year	0.506	—	—	—
PIRCHE-II HLA-C^§^	≤1 year	0.192	—	—	—
	>1 year	0.503	—	—	—
PIRCHE-II HLA-class I^§^	≤1 year	0.689	—	—	—
	>1 year	0.516	—	—	—
PIRCHE-II HLA-DR^§^	≤1 year	0.130	—	—	—
	>1 year	0.115	—	—	—
PIRCHE-II HLA-DQ^§^	≤1 year	**0.033**	**1.038**	**1.011–1.066**	0.011*
	>1 year	**0.008**	**1.023**	**1.008–1.038**	0.025*
PIRCHE-II HLA-class II^§^	≤1 year	0.027	—	—	—
	>1 year	0.018			
Total PIRCHE-II^§^	≤1 year	0.130	—	—	—
	>1 year	0.080			
Simultaneous pancreas/kidney transplantation (SPKT)	≤1 year	0.015*	1.020	0.996–1.043	0.765
	>1 year	0.978	—	—	—
Preformed DSA	≤1 year	0.297	—	—	—
	>1 year	0.894	—	—	—
Interaction (SPKT x preformed DSA)	≤1 year	<0.001*	2.361	0.658–8.468	0.188
	>1 year	0.030*	**2.782**	**1.061–7.294**	0.037*
Recipient age	≤1 year	0.235	—	—	—
	>1 year	0.078	1.003	0.996–1.011	0.403
Donor age	≤1 year	0.001*	**0.965**	**0.943–0.988**	0.003*
	>1 year	0.013*	0.987	0.973–1.002	0.189
T-cell depleting induction	≤1 year	0.014*	0.516	0.234–1.137	0.101
	>1 year	0.821	—	—	—
Type of calcineurin inhibitor (Ciclosporin)	≤1 year	0.216	—	—	—
	>1 year	0.004*	**2.440**	**1–464–4.069**	<0.001*

^§^ *P*-values were adjusted for multiple comparisons using the Bonferroni correction, with a corrected significance level of 0.0056 (0.05/8) applied to the analyses involving the different PIRCHE-II scores. *P*-values ≤ 0.00625 are considered statistically significant.

* statistically significant.

## Discussion

A well-established correlation has been suggested in kidney transplantation between a higher number of HLA epitope mismatches [17] and an increased risk of developing dnDSA associated with AMR and allograft loss [18–21]. Lachmann et al. revealed in their paper a strong correlation between the total PIRCHE-II score (considering HLA-locus A, HLA-locus B, HLA-locus C, HLA-locus DR, HLA-locus DQ) and an increased risk of development dnDSA, primarily directed against HLA-DQ, followed by HLA-DR, HLA-A, and HLA-B mismatches. This was confirmed in subsequent studies [22, 23].

In contrast to the investigations carried out in a kidney transplantation cohort, the PIRCHE-II scores’ prognostic value in predicting dnDSA development and graft outcomes following other solid organ transplantations is not well studied. Particularly in pancreas transplantation, data regarding the relevance of PIRCHE-II scores is scarce. Based on suggested risk assessment according to the recently published First World Consensus Conference on pancreas transplantation [1, 2], less importance has been attributed to HLA mismatching and preformed DSA. In this context, our study aims to evaluate risk factors for developing dnDSA among SPKTRs. To the best of our knowledge, this study represents the first effort to investigate the influence of the PIRCHE-II scores, adjusted for both HLA class I and II, and HLA locus-specific, on the development of dnDSA in a cohort of SPKTRs.

Firstly, our results indicate that, despite SPKTRs having fewer preformed DSA and lower median total PIRCHE-II scores, there was a higher incidence of dnDSA against HLA class I and II in the first post-transplant year compared to KTRs. Notably, dnDSA were predominantly directed against the HLA-locus DQ, consistent with previous studies [24]. Several factors contribute to this finding. The pancreas allograft is a highly immunogenic organ, and its beta cells can prompt a strong alloimmune response, contributing to a higher incidence of dnDSA development. Pancreatic inflammation and injury, common in the early post-transplant period, can further activate alloreactivity, leading to the development of dnDSA as the immune system reacts to the inflamed or injured pancreatic tissue. Although SPKTRs receive more intense induction immunosuppressive therapy, rapid steroid withdrawal might not be suitable for all SPKTRs and allow DSA development. Given the observed differences in alloreactivity during the early post-transplant period, it is particularly crucial to study the impact of PIRCHE-II scores on dnDSA formation in SPKTRs. To reduce the potential risk of overestimation of dnDSA with an MFI cut-off of 500, all dnDSA were analyzed individually by a specialist in transplantation immunology in a blinded fashion. Here, 1) analyzing the pattern of single-bead reactivity and comparing it to the HLA typing of the donor, 2) investigating for epitope specificity to determine alpha chain binding antibodies in the setting of HLA-DQ and DP, 3) determining unspecific reactivity by comparing the pattern of reactivity to lot-specific reactivity patterns in non-immunized males that are continuously tracked in our transplant laboratory, and 4) incorporating the reactivity to the recipient’s own HLA antigens was applied to reduce overestimation.

Secondly, regarding the risk factors associated with the development of dnDSA in SPKTRs, the presence of preformed DSA and younger donor age were independently associated with an increased risk. Interestingly, total PIRCHE-II scores, PIRCHE-II scores per HLA class, and PIRCHE-II scores per HLA locus were not independently associated with an increased risk for the development of dnDSA. However, PIRCHE-II scores for HLA class II, particularly HLA-locus DQ, may predict the development of dnDSA against HLA class II, although the sample size in our analysis was not sufficient to show this association independently upon multivariate analysis. This finding aligns with the observation that dnDSA against HLA-locus DQ exhibited the highest incidence among all HLA loci. Conversely, the lack of association for HLA class I is likely attributable to the low incidence of dnDSA against HLA class I and the small sample size of our cohort. Nonetheless, our results highlight other factors associated with the development of dnDSA that should be considered in future studies when evaluating the predictive and additive value of PIRCHE-II scores.

Notably, preformed DSA increased the risk of dnDSA development among SPKTRs but not KTRs. Factors associated with SPKT, preformed DSA, and the combination of both may likely explain this elevated risk of dnDSA development. Factors associated with SPKT include 1) the transplantation of two organs, which presents more allo-antigens compared to kidney transplantation alone, and 2) the pancreas as a highly vascularized organ, that may provoke a stronger alloimmune response compared to the kidney alone. Factors associated with preformed DSA include 1) the presence of an already activated immune system with presence of memory B cells, that may get stimulated by the increased antigen load in SPKT, and 2) a high number of shared HLA-epitopes, that contribute to the potential of HLA antibodies cross-reacting with other HLA antigens [25]. Our results suggest, that the combination of SPKT and preformed DSA is decisive for the increased risk of dnDSA development. 1) Preformed DSA may precipitate subclinical or clinical TCMR and AMR, particularly in the pancreas allograft, which can further induce an inflammatory microenvironment that may stimulate antigen presentation and immune activation, leading to dnDSA development. 2) The rapid steroid withdrawal in SPKTRs may also be critical in cases with preformed DSA, facilitating this inflammatory microenvironment that may allow the formation of dnDSA. Organs from younger donors tend to have higher immunogenicity due to increased expression of HLA antigens and costimulatory molecules. Additionally, the presence of more active dendritic cells and other antigen-presenting cells, coupled with increased cellular proliferation, can enhance antigen exposure and the recipient’s immune activation, thereby increasing the risk of DSA formation.

Thirdly, when considering the entire cohort of SPKTRs/KTRs, simultaneous pancreas/kidney transplantation did not independently increase the risk of developing dnDSA. However, our data demonstrated an association between HLA epitope mismatching and dnDSA development, consistent with the literature [15]. Unlike previous studies, we observed the most pronounced association between PIRCHE-II scores for HLA-locus DQ and the development of dnDSA against HLA-class II. Ladowski et al. reported similar results but primarily focused on PIRCHE-II for HLA class II, especially HLA-DQB mismatches [24]. The concept of HLA epitope mismatch load and the impact of dnDSA is most clearly shown for HLA-locus DQ. It remains unknown whether the number of HLA epitope mismatches or the increased likelihood of more immunogenic HLA epitopes contributes to this increased risk [26]. Current evidence suggest HLA-DQ combinations that are more immunogenic than others [27].

To our knowledge, we are among the first to include PIRCHE-II for both HLA-DQB and HLA-DR in the SPKTRs population and demonstrate their role in predicting dnDSA formation against both HLA class I and II. Additionally, Chaigne et al. demonstrated in a cohort of pancreas recipients that the formation of anti-HLA class I antibodies was unrelated to PIRCHE-II scores. In contrast, the development of anti-HLA class II antibodies was influenced by PIRCHE-II scores [28]. We did not observe an association between PIRCHE-II scores for HLA-locus C, which may explain the lack of significance for total PIRCHE-II scores in our cohort. This finding aligns with previous studies, such as those by Lachmann et al., who also considered HLA-A, B, C, DR, and DQ when calculating the PIRCHE-II score [15]. Thus, our observation highlights the lack of association with the PIRCHE-II score for HLA-C, which can potentially contribute to misleading interpretations of total PIRCHE-II scores. Moreover, we observed an increased risk of dnDSA with the use of ciclosporin and lower donor age [29]. These findings from our multivariate analysis are significant because the two most cited studies, by Lachmann et al. [15] and Unterrainer et al [30], are based on data from patients primarily under ciclosporin-based immunosuppression and with incomplete recipient and donor typing. These earlier studies may not fully reflect the current state of transplantation practices.

Our study possesses several strengths. First, it stands as one of the first analyses focusing on the development of dnDSA in SPKTRs compared to KTRs. Second, we included a well-characterized cohort of SPKTRs spanning over a decade, adhering to a standardized immunosuppressive protocol without the use of ciclosporin, and maintained close clinical and immunological post-transplant monitoring, thus providing high data density. Third, our study explored, for the first time in SPKTRs, the total PIRCHE-II scores, PIRCHE-II scores per HLA-class, and PIRCHE-II scores per HLA-locus. Yet, there are also limitations warranting consideration. Most importantly, the retrospective study design, small sample size and the single-center bias concerning allograft allocation, immunosuppressive strategy based on a steroid-free immunosuppression regimen in SPKTRs. Our study also relied on imputed high-resolution HLA alleles for the PIRCHE-II calculation, which could potentially influence our results due to errors in the imputation. The population in our study was predominantly Caucasian and a recent study has suggested that the potential difference in PIRCHE-II score association with dnDSA development in this setting would be minimal [31].

In summary, SPKTRs exhibit a higher incidence of *de novo* dnDSA in the first year post-transplantation, which is not linked to an increased HLA-epitope mismatch load. The correlation with preformed DSA indicates a higher immunologic risk, particularly under a steroid-free regimen, favoring dnDSA development. Over the long term, a high HLA-epitope mismatch load for HLA locus DQ is similarly crucial for dnDSA development in both SPKTRs and KTRs. The lack of association between the total PIRCHE-II score, PIRCHE-II scores for HLA classes, and other HLA loci suggests that these biomarkers should at the moment not be used for risk stratification post-transplantation.

## Data Availability

The raw data supporting the conclusions of this article will be made available by the authors, without undue reservation.

## Footnotes

1
https://www.pirche.org

## Supplementary Material

Supplementary Figure S1
**(A)** Development of TCMR between SPKTRs (red) and KTRs (green; *p* = 0.415) with 14.2% vs. 10.6% at 10 years post-transplant, respectively. **(B)** Development of ABMR was comparable between SPKTRs (red) and KTRs (green; p = 0.447) with 8.1% vs. 11.1% at 10 years post-transplant, respectively.

Supplement Figure S2 Development of *de novo* DSA against HLA-class II DP was comparable between SPKTRs (red) and KTRs (green; *p* = 0.184) with 8.7% vs. 5.8% at 10 years post-transplant, respectively. *De novo* DSA at 1 year post-transplantation were detectable in 1.4% of SPKTRs vs. 0.8% of KTRs (*p* = 0.606).

## Abbreviations

ABMR, antibody-mediated rejection; BMI, body mass index; CNI, calcineurin inhibitor; dnDSA, *de novo* donor-specific anti-HLA-antibodies; eGFR, estimated glomerular filtration rate; HbA1c, hemoglobin A1c; KT, kidney transplantation; KTRs, kidney transplant recipients; MFI, mean fluorescence intensity; MMF, mycophenolate mofetil; MPA, mycophenolic acid; NGS, next-generation sequencing; PIRCHE, Predicted Indirectly ReCognizable HLA Epitopes; PT, pancreas transplantation; PTA, pancreas transplantation alone; SPK, simultaneous pancreas/kidney transplant; SPKTRs, simultaneous pancreas/kidney transplant recipients; SSO, sequence-specific oligonucleotide; SSP, sequence-specific primer; TCMR, T-cell mediated rejection.
