# Supplementary figures and images for: Assessing the Predictive Power of PIRCHE-II Scores for the Development of De Novo Donor-Specific Antibodies After Simultaneous Pancreas-Kidney Transplantation
Source: Transpl Int. 2024 Dec 18;37:13720. doi: 10.3389/ti.2024.13720 (PMC11688186; doi:10.3389/ti.2024.13720)

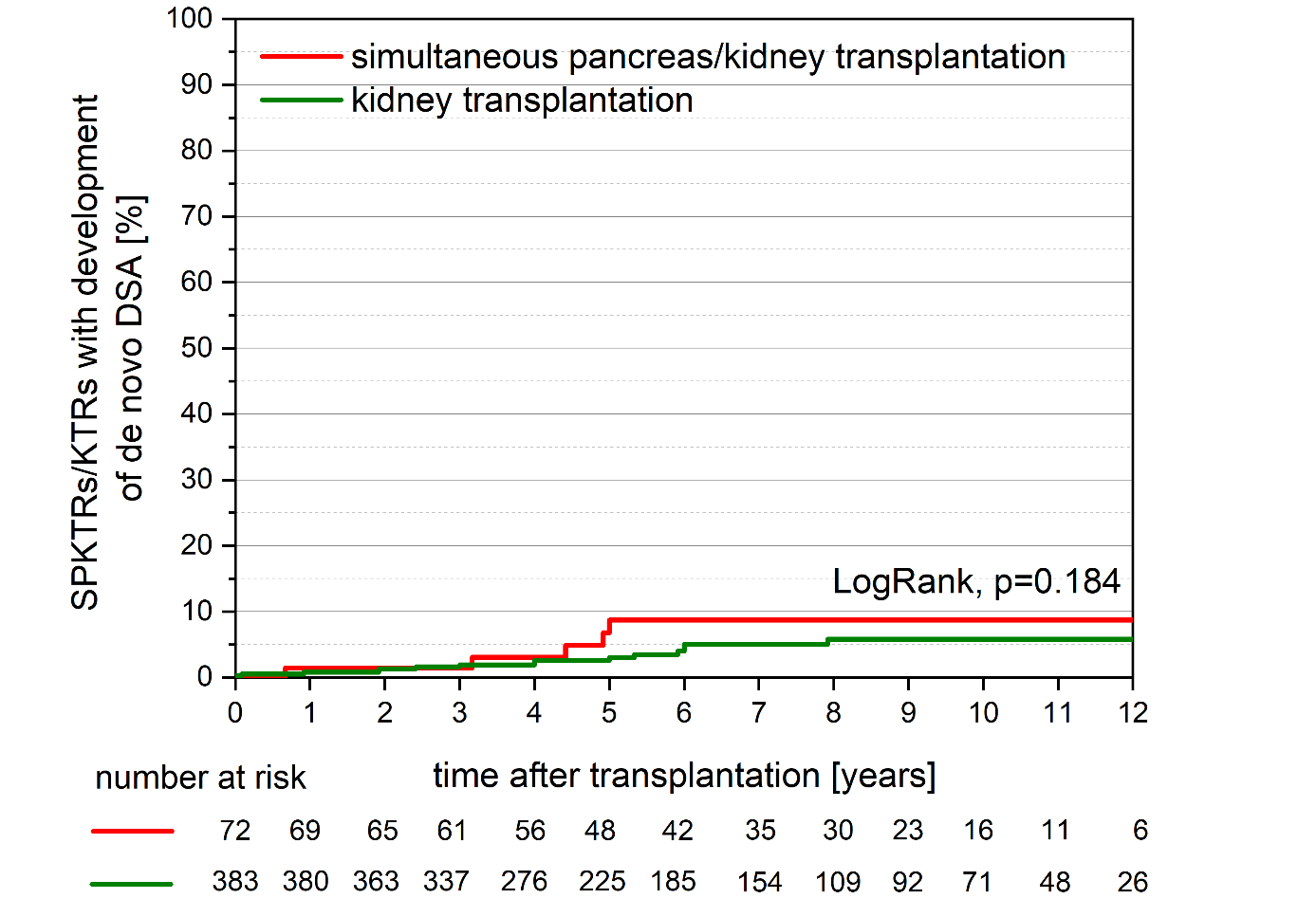

Supplement: Supplementary file 1 [file Image3.TIF]

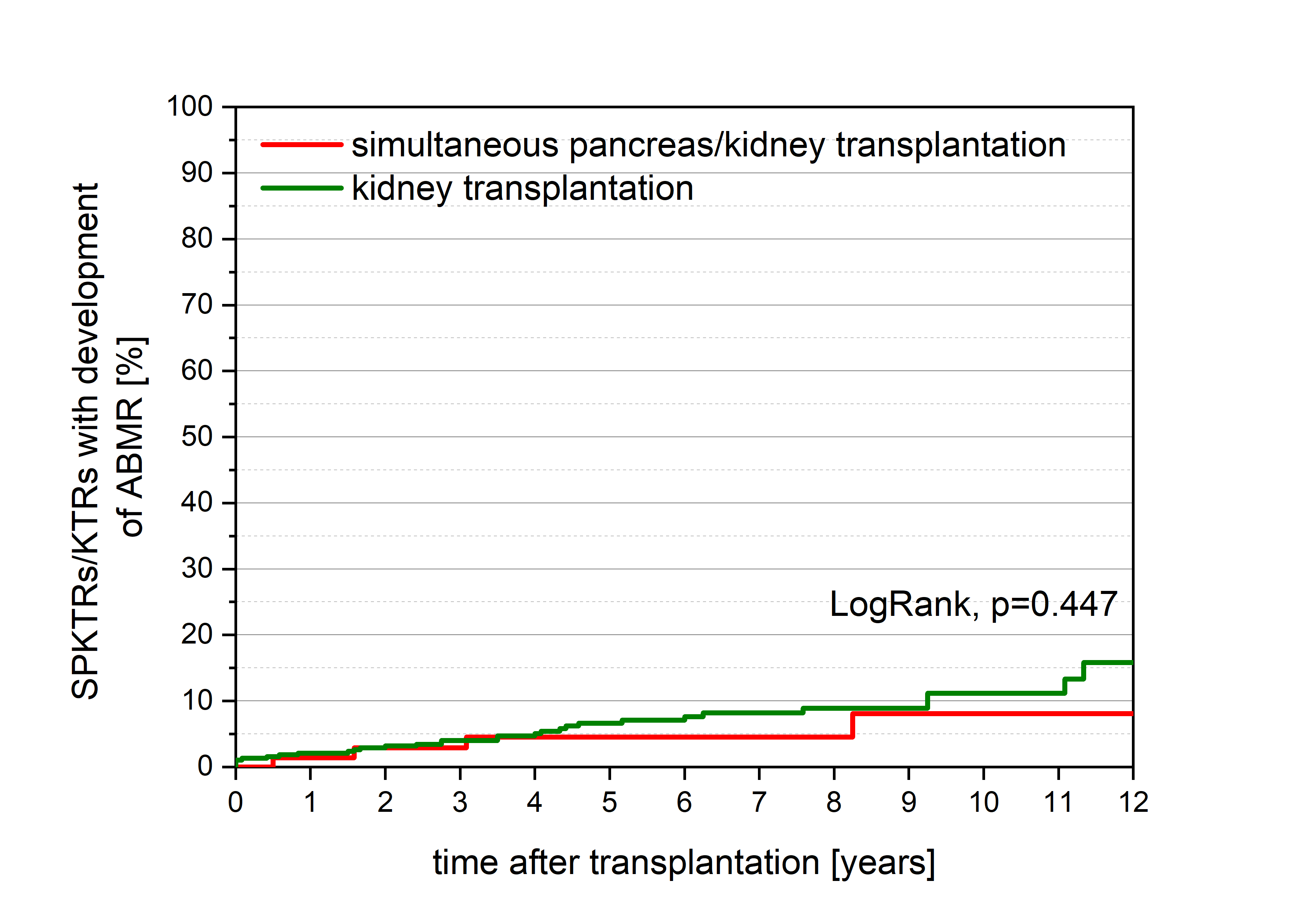

Supplement: Supplementary file 2 [file Image2.TIF]

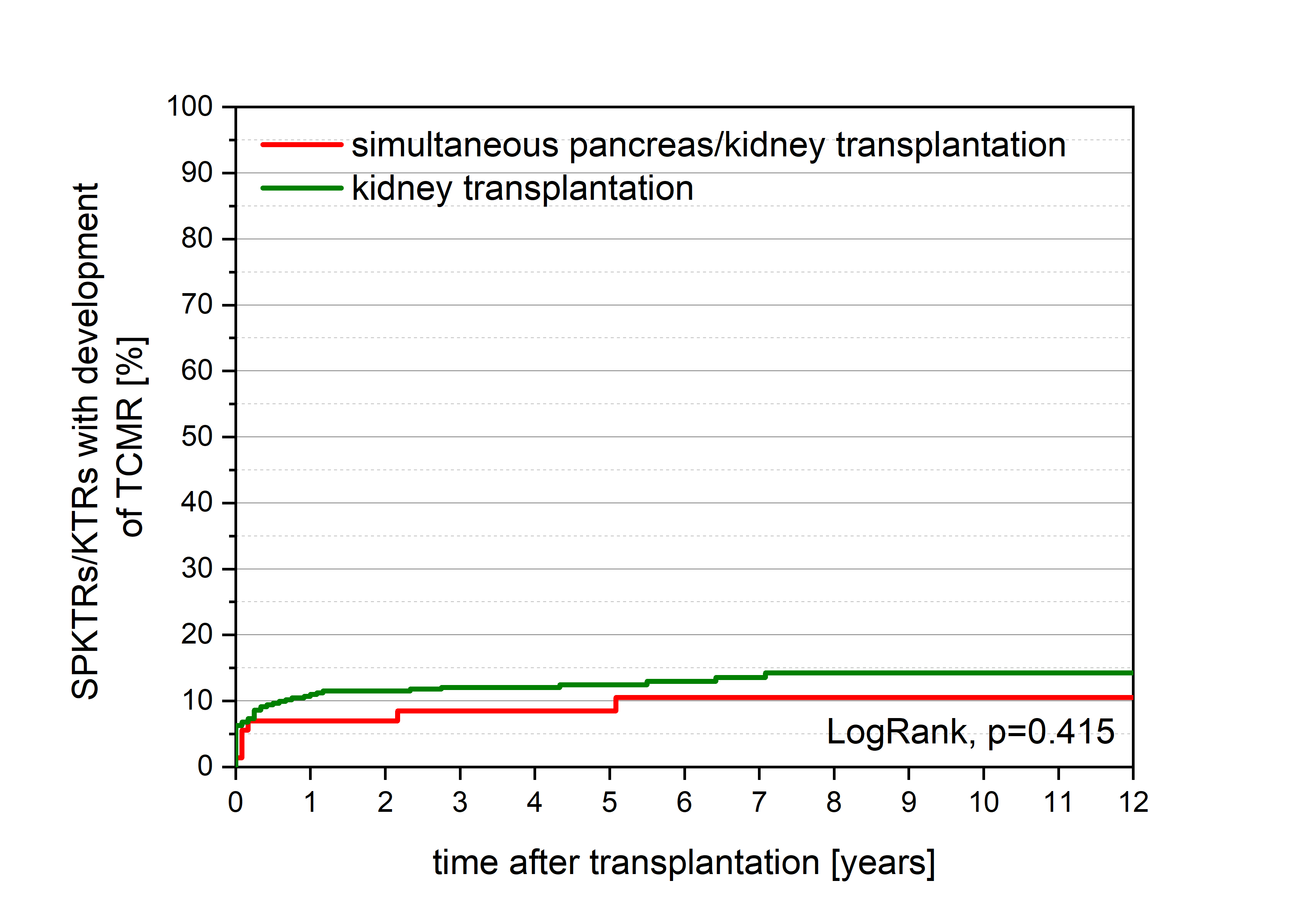

Supplement: Supplementary file 3 [file Image1.TIF]
